# Annotations

**Published:** 1894-02-17

**Authors:** 


### ANNOTATIONS.

### A Reverend Nottingham Surgeon.

The Rev. Henry Seymour, M.A., Chairman of the Weekly Board of the Nottingham Hospital gave at the annual meeting of the hospital committee the other day, a *résumé* of the history of surgery which it would have taxed the intelligence of almost any surgeon in these islands to have equalled for interest and comprehensiveness. From the dawn of surgery in the infancy of the world, across the ages, sometimes illuminated by the light of intelligence, but oftener murky with the thick darkness of ignorance and prejudice, the reverend amateur surgeon drove his skiff of appreciative criticism until he reached the benignly scientific shores of the anatomic, anæsthetic, and antiseptic surgery of our own times. The monks of the middle ages, he tells us, were excellent nurses, but bad surgeons. They insisted that the shedding of blood was contrary to religion. He gives Celsus' description of a model surgeon, one sentence of which makes us thank our stars that we live in the days of anæsthetics. Says Celsus: "The surgeon should be intrepid, and his sensibility should be such that, determined to cure his patient, he should be unmoved by his cries, and should neither hasten the operation nor cut less than he ought. Mr. Seymour passes over John Hunter, which an actual surgeon would hardly have done. To anæsthetics he does full justice, and to antiseptics also. On this latter development of surgery his testimony is almost more eloquent than he himself is aware of. He tells us, for instance, that the house surgeon had informed him that there had been in the hospital recently a large number of very severe cases of compound fracture, and that in every case the fractured limb had been saved. In most, or all of these cases, immediate amputation would have been resorted to as a matter of course twenty or twenty-five years ago. We congratulate the Rev. Henry Seymour on the excellence of his historical sketch, and the Nottingham Hospital on the intelligence of its chairman.

### The Drink Cure at Dundee.

Seventeen persons were placed under a particular treatment for drunkenness at Dundee on the 5th of January of the present year, and the treatment was finished and reported upon in the *Scotsman* newspaper of February 3rd. The whole experiment had been made and completed within a month. The committee, of which Bailie D. S. Smith was chairman, and Mr. James Thomson, Burgh Fiscal, honorary secretary, report that "in all the cases the drink crave had been entirely eradicated, and a distinct loathing for intoxicating liquors had taken its place." There need be no doubt entertained as to the perfect good faith and good intentions of Bailie Smith, Mr. James Thomson, and their fellow committee men. But we suggest to them two things: First, that they were not more competent to deal with the question of the use and value of a medicinal substance than they were to undertake the building of the Forth Bridge; and, secondly, that they have proved their complete inability to comprehend the real nature of the problem they undertook to solve by announcing that they are entirely satisfied with a month's trial of only seventeen cases. A physiologist who really understands this kind of medical experimentation would not have been satisfied with less than a thousand cases and a five years' trial. What is more common than for the most notorious drunkards to remain sober not for one month, but for three, and that without any treatment at all? And what can be more certain than that the average drunkard will loathe and abominate even the very name of drink for a certain number of days, and even weeks, after a bad drinking bout? This is the appeal which plain facts and common experience make to common sense. It has fallen to the lot of the present writer during the past three years to give a prolonged and impartial consideration to these attempts on the part of licensed or unlicensed practitioners of medicine to carry their wares to the market of the unlearned. In no case has he seen anything like honesty or square dealing on the part of the irregular practitioners. On the contrary, he has found the whole thing knavish in root and branch. Public morality, as well as public health, demands the suppression of these practices. The medical profession, which is the natural guardian of the public health, is fast making up its mind that quackery must be extinguished. This is right. A bold and determined campaign should be entered upon, and the knaves who lie and cheat, because they find the public so easy to deceive, must be taught by prison discipline that "the way of transgressors is hard" in an intelligent community.

### Are Anarchists Lunatics?

That Anarchists as a matter of fact are lunatics is as obvious to common sense as that a dog is mad when he rushes at friends and foes indiscriminately, and with frantic teeth endeavours to tear them to pieces. The point of our question is this: Are European jurists prepared to pronounce Anarchists technically insane; and will legislatures enact statutes for their certification and restraint? The medical mind foresees an epidemic of Anarchism in the near future, and its natural instinct is to look about for preventive measures. The only preventive measures of any avail to avert severe epidemics of contagious diseases in cattle have been the killing of the infected cattle in the very earliest stages of the malady. In the case of human beings, the very early detection of cases and their prompt isolation have alone been efficient. Our first business is to secure the enactment of a statute or statutes declaring Anarchists and inciters to anarchy "insane." When that has been done, competent investigators must be set to work to find out every possible British or foreign Anarchist or inciter to anarchy which our own country may harbour. These must then all be locked up in one or more criminal lunatic asylums, and there medically treated until such as are capable of cure are thoroughly cured; the remainder will be kept under restraint for life. Can we induce any leading member of Parliament to take this matter up, and to get his Bill through before anarchism attains to epidemic dimensions in our own country? We would fain insure the locking of the stable door before the steed is stolen, and not, as in France, after.

